# Exploring antibody repurposing for COVID-19: beyond presumed roles of therapeutic antibodies

**DOI:** 10.1038/s41598-021-89621-6

**Published:** 2021-05-13

**Authors:** Puneet Rawat, Divya Sharma, Ambuj Srivastava, Vani Janakiraman, M. Michael Gromiha

**Affiliations:** 1 Protein Bioinformatics Laboratory, Department of Biotechnology, Bhupat and Jyoti Mehta School of Biosciences, Indian Institute of Technology Madras, Chennai, Tamil Nadu 600036 India; 2 Infection Biology Lab, Department of Biotechnology, Bhupat and Jyoti Mehta School of Biosciences, Indian Institute of Technology Madras, Chennai, Tamil Nadu 600036 India

**Keywords:** Computational biology and bioinformatics, Immunology

## Abstract

The urgent need for a treatment of COVID-19 has left researchers with limited choice of either developing an effective vaccine or identifying approved/investigational drugs developed for other medical conditions for potential repurposing, thus bypassing long clinical trials. In this work, we compared the sequences of experimentally verified SARS-CoV-2 neutralizing antibodies and sequentially/structurally similar commercialized therapeutic monoclonal antibodies. We have identified three therapeutic antibodies, Tremelimumab, Ipilimumab and Afasevikumab. Interestingly, these antibodies target CTLA4 and IL17A, levels of which have been shown to be elevated during severe SARS-CoV-2 infection. The candidate antibodies were evaluated further for epitope restriction, interaction energy and interaction surface to gauge their repurposability to tackle SARS-CoV-2 infection. Our work provides candidate antibody scaffolds with dual activities of plausible viral neutralization and immunosuppression. Further, these candidate antibodies can also be explored in diagnostic test kits for SARS-CoV-2 infection. We opine that this in silico workflow to screen and analyze antibodies for repurposing would have widespread applications.

## Introduction

COVID-19, caused by the novel coronavirus SARS-CoV-2, has emerged to be a global pandemic affecting over 46 million people worldwide so far (https://covid19.who.int/). The highly contagious virus SARS-CoV-2 belongs to the *betacoronavirus* genus of the *Coronaviridae* family^1^. It is an enveloped single-stranded positive-sense RNA virus with a genome size of ∼ 30,000 base pairs^2,3^. The viral genome encodes for 4 structural and 16 non-structural proteins. The spike structural protein (S), in particular, plays a vital role in fusion, entry, and transmission into the host cells. The S protein contains an N-terminal S1 subunit, responsible for the virus-receptor binding and a C-terminal S2 subunit, responsible for virus-cell membrane fusion^4,5^. The receptor-binding domain (RBD) in the S1 subunit of spike protein allows entry into the host cell via attachment to the angiotensin-converting enzyme 2 (ACE2) receptor^6^. Therefore, spike protein is currently a major therapeutic target for evolving interventions for COVID-19^7–10^.

The combined efforts of the scientific community have substantially improved our understanding of the virus and the disease pathology in a short period. There have been several attempts to identify therapeutics for SARS-CoV-2 infection using experimental and computational approaches. Initial studies on COVID-19 suggested the importance of specific drugs such as ivermectin^11^, a combination of lopinavir, oseltamivir and ritonavir^12^; Remdesivir^13^ and hydroxychloroquine (HCQ)^14^ as potential ones against COVID-19. However, Remdesivir is the only drug currently approved by the FDA (https://www.fda.gov/news-events/press-announcements/fda-approves-first-treatment-covid-19). A large-scale experimental study on 12,000 compounds for drug repurposing showed a set of 13 compounds to be effective against SARS-CoV-2^15^. Other computational approaches included structure-based virtual screening^16^ and virus-host interactions network analysis to identify potential anti-SARS-CoV-2 repurposable drugs^17^. A recently developed online platform CoVex integrated virus-human protein interactions, human protein–protein interactions, and drug-target interactions to explore the host interactome and identification of drug(s) related to SARS-CoV-2^18^. Few antibody therapies including Etesevimab, REGEN-COV (Casirivimab and Imdevimab) and Bamlanivimab are authorized for emergency use to treat mild-to-moderate COVID-19 cases (by March 2021; https://www.fda.gov/drugs/coronavirus-covid-19-drugs/coronavirus-treatment-acceleration-program-ctap)^19^.

As an alternate strategy for immediate relief in seriously ill patients, convalescent sera from recovered COVID-19 patients, supposedly rich in anti-SARS-CoV-2 antibodies, is in use. The antibodies present in the convalescent sera have been isolated and studied in detail for binding to spike protein, viral neutralization and cross-reactivity with spike proteins of SARS-CoV-2^20,21^. S protein regions comprising RBD have shown to elicit multiple neutralizing antibodies that can neutralize SARS-CoV-2 by targeting different epitopes^20,22^. Raybould et al.^23^ recently developed a coronavirus antibody database, “CoV-AbDab”, with curated data on published antibodies and nanobodies related to different coronavirus strains. Taken together, in the development of clinical interventions against SARS-CoV-2, neutralizing antibodies play a significant role. On the other hand, pharmaceutical agencies supported this strategy and developed two neutralizing mAbs, VIR-7831 and VIR-7832, as a potential therapeutic intervention (https://www.nature.com/articles/d43747-020-01115-y). In addition, a cocktail of two neutralizing monoclonal antibodies (mAbs) are being tested in phase 2/3 trials for the treatment and prevention of SARS-CoV-2 infection.

In this work, we have attempted antibody repurposing with a goal to identify therapeutic antibodies that are already approved or in clinical trials for potential cross-reactivity towards neutralization of the SARS-CoV-2. Sequences of 190 neutralizing antibodies specific to SARS-CoV-2 spike protein^23^ were compared with the dataset comprising 552 therapeutic antibodies^24^. Finally, four antibody pairs, including four neutralizing antibodies and three therapeutic antibodies, were selected and further scrutinized by a comprehensive analysis that includes (1) docking to the spike protein; (2) epitope overlaps; (3) interaction energy; (4) interaction area; (5) common contacts for therapeutic antibodies (with native target and spike protein), to effectively estimate the potential binding and a plausible replication of SARS-CoV-2 neutralization activity. Interestingly, the selected candidates, Tremelimumab, Ipilimumab and Afasevikumab are anti CTLA4 and anti IL17A antibodies. Severe SARS-CoV-2 infection is consistent with elevated levels of CTLA4 and IL17A. We posit that these candidate antibodies offer promise as ready to use and/or with the potential for better developability for COVID-19 management. The novel workflow presented here can be implemented for antibody repurposing for several other pathologies.

## Materials and methods

### Dataset preparation

We collected a set of 190 antibody sequences from CoV-abDab database^23^ using the following criteria: (1) Source of the antibody should be B cells from convalescent individual(s); (2) variable region sequence information of the heavy chain (V_H_) and light chain (V_L_) should be available; (3) neutralization activity against the SARS-CoV-2 should have been demonstrated and (iv) binding to the RBD region of the spike protein should have been confirmed. Similarly, a dataset of 552 therapeutic antibody sequences was collected from the Thera-SAbDab database^24^ for which V_H_ and V_L_ sequence information was available.

Regions considered as plausible epitopes in the RBD region of spike protein, regarded as “known epitope dataset”, were obtained from the crystal structures of neutralizing antibody-spike protein complexes deposited in Protein Data Bank, PDB^25^ and the information available in the literature^22,26^. The epitope residues in the spike protein were identified by setting a cutoff of 4 Å for distance with the antibody residues in a spike protein–antibody complex^27^.

### Comparative analysis of antibody sequences and structures

**Figure 1:**
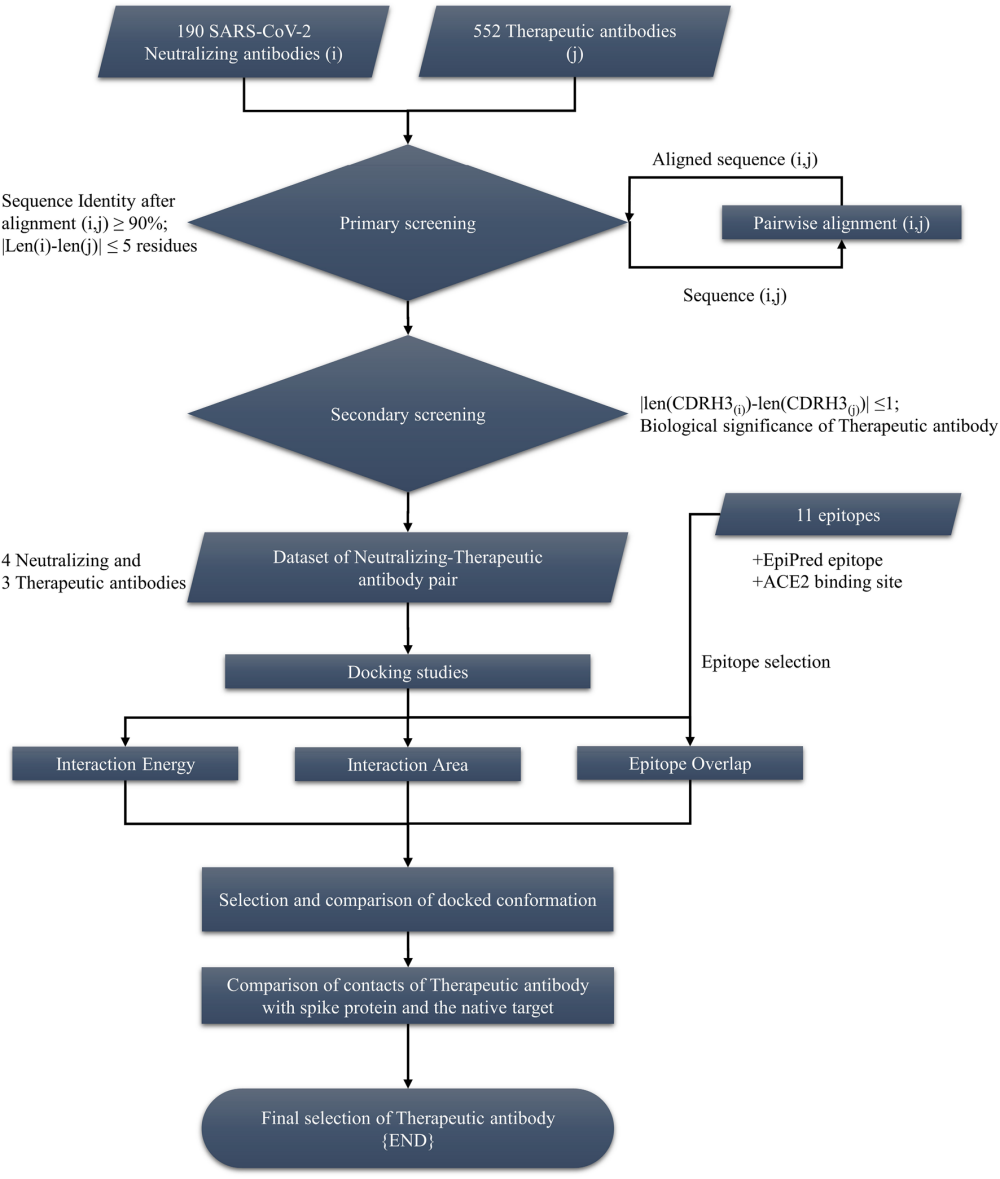
Workflow illustrating the steps followed to screen therapeutic antibodies for potential repurposing against SARS-CoV-2.

The sequences of the SARS-CoV-2 neutralizing and therapeutic antibodies were compared by generating pairwise alignment for all 104,880 combinations (552*190) using Biopython^28^ as shown in Fig. 1. The maximum percent identity for each neutralizing-therapeutic antibody pair was calculated by dividing the total number of matches at the respective position in the aligned sequence with the length of the shorter antibody sequence (Eq. 1). The maximum identity was selected to accommodate as many antibodies as possible in the primary screening. The final cutoff of 90% was selected for screening and the antibody pairs with the length difference of more than five residues were removed.

1 $$\% Identity= \frac{Number\, of\, matched\, residues\, for\, each\, position\, in\, alignment*100}{\mathrm{Min}(length\, of\, antibodies\, in\, pair\, \left({V}_{H}+{V}_{L}\right))}$$

The light and heavy chain sequences were annotated using ANARCI tool^29^. The sequences and regions of third complementarity-determining regions for heavy chain (CDRH3) and light chain (CDRL3) were extracted based on the IMGT numbering scheme^30^. The antibody pairs were visualized in JalView^31^ after aligning with MAFFT^32^. Antibodies without structure information were modelled computationally using an automated antibody modelling pipeline called “ABodyBuilder”^33^. To identify the structural similarity between any two antibody structures, we superimposed them and estimated the root mean square deviation (RMSD) for the aligned regions.

### Epitope prediction and comparison

The epitopes on the spike protein were predicted using a structure-based method called “EpiPred” for each neutralizing and therapeutic antibody^34^. The unique epitopes on the RBD region were selected from the “known epitope dataset” based on the following conditions: (1) least overlap (< 80% identity cutoff) with other regions considered to be epitopes to reduce redundancy, (2) epitope length is close (± 4 amino acid residues) to the average epitope length on the spike protein (~ 22 amino acid residues, from “known epitope dataset”) to avoid inclusion of too short and/or long epitopes and (3) overlap with the ACE2 binding site for potential virus neutralization ability. This procedure yielded three potential epitopes, CB6, S2H14 and S2H13 (Table S5, Figure S4).

### Docking studies and antibody-SARS-CoV-2 spike protein complex analysis

We have carried out molecular docking using ClusPro 2.0^35^ and generated the structures (30 conformations each) of SARS-CoV-2 spike protein complexed with both SARS-CoV-2 specific neutralizing antibodies and therapeutic antibodies. The optimal docked conformation was selected for each SARS-CoV-2 spike protein-antibody complex based on (1) binding to unique epitopes in “known epitope dataset” (maximum overlap), (2) interaction energy and (3) interaction area. The interaction energies of the docked structures at chosen conformations were calculated using FoldX^36^. Accessible surface areas of the RBD region in the spike protein (S_E_), antibody $\left({S}_{H+L}\right)$ as well as the entire complex (${S}_{complex}$) were calculated by rolling a water molecule of radius 1.4 Å on the protein/complex surface. Further, the area of the interface (${S}_{interface}$) was calculated using Eq. (2).

2 $${S}_{interface}=({S}_{E}+{S}_{H+L})-{S}_{complex}$$

### Comparative assessment of contacts of therapeutic antibodies with their native targets and SARS-CoV-2 spike protein

The contact pattern between the amino acid residues of these therapeutic antibodies and their respective native targets was compared with contacts of the therapeutic antibody—SARS-CoV-2 spike protein complex obtained from the docking studies. The epitope and paratope residues are compared accordingly for each therapeutic antibody to assess the similarity in their mode of binding and common interactions, which in turn will help in understanding the basis for therapeutic antibody binding to RBD region of SARS-CoV-2 spike protein.

## Results

### Screening of the therapeutic antibodies

The first step in identifying potential antibodies for repurposing is screening therapeutic antibodies similar to known SARS-CoV-2 neutralizing antibodies. The screening was done in two stages:

In the primary screening, variable region of heavy chain and light chain (V_H_ and V_L_) sequences of the antibodies were combined into a single sequence in order to pick up full-length antibody pairs, which were used further to compute the sequence identity for all combinations of neutralizing-therapeutic antibody pairs (552*190 combinations). The maximum percent identities of the antibody pairs, obtained from the pairwise sequence alignment, were grouped based on the descending identity cut offs, ranging from 95 to 85%, as shown in Figure S1. The number of antibody pairs increased exponentially with a decrease in cutoff. We selected the optimal number of antibodies at 90% max identity cut off (containing 88 antibody pairs with 37 unique neutralizing antibodies and 41 unique therapeutic antibodies) to allow multiple levels of screening downstream without exhausting all the antibodies and to facilitate manual screening. We also removed antibody pairs with a high difference in sequence length (at least five residues) in this primary screening. Antibodies selected after primary screening are given in Table S1.

The secondary screening of the antibodies consisted of two levels: (1) difference in the length of the third complementarity-determining regions for heavy chain (CDRH3) and (2) feasibility of the selected therapeutic antibody acting as a neutralizing antibody for SARS-CoV-2. Antibodies generally have canonical structures for the 5 CDRs (except CDRH3). CDRH3 regions show high diversity in sequence length, composition and loop structure^37^. These regions are also the major contributors to antibody specificity^38^. We have attempted to limit the diversity of the CDRH3 loop by selecting only the CDRH3 sequences of similar length (length difference of ± 1 amino acid residue; Table S2). The specificity of the CDRH3 is assessed using docking studies and interaction energy calculations. A similar analysis was also done for the CDRL3, where most of the CDRL3 sequences were of equal length. The final dataset after all these screening procedures contained 11 neutralizing-therapeutic antibody pairs (7 unique neutralizing antibodies and 10 unique therapeutic antibodies). Nurulimab is one of the selected therapeutic antibodies, similar to Ipilimumab, with a sequence identity of > 99% (Table S3a). It was removed from the dataset to reduce redundancy and the selected candidate therapeutic antibodies were further assessed for their biological significance (Table 1).

**Table 1:** List of shortlisted therapeutic antibodies and their specifics.

Therapeutic mAbs	Length (aa) (V_H_ + V_L_)	Clinical trial status (Jan-2020)	Target	Clinical condition(s) (active/approved)	Year proposed
**Afasevikumab**	**231**	**Phase-I**	**IL17A**	**Inflammation**	**2015**
Daratumumab	229	Approved	CD38	AL amyloidosis; cancer	2009
Denosumab	230	Approved	TNFSF11	Cancer	2005
Enapotamab	224	Phase-II	AXL	Solid tumors	2017
**Ipilimumab**		**Approved**	**CTLA4**	**Cancer**	**2005**
Marstacimab		Phase-III	ERBB2	Haemophilia	2013
Ofatumumab	229	Approved	MS4A1	Cancer	2005
Simlukafusp		Phase-II	FAP	Cancer	2019
**Tremelimumab**	**232**	**Phase-III**	**CTLA4**	**Cancer**	**2005**

Highlighted antibodies were chosen as promising candidates. Further comprehensive analysis was carried out on these candidate antibodies to gauge their potential use in COVID-19 management.

### Biological significance of the candidate therapeutic antibodies

We have further assessed the 9 selected therapeutic antibodies (Table 1) for their biological significance and feasible use in treating SARS-CoV-2 infection. Most of the strategies currently available for drug repurposing for COVID-19 target human proteins involved in the host–virus interaction network due to limited options available for targeting the viral proteins^17,18^, which might lead to adverse effects. Therefore, a careful selection of therapeutic antibodies is important. We found that Afasevikumab, Ipilimumab and Tremelimumab are of special interest for SARS-CoV-2 infection (highlighted in Table 1). Among the three therapeutic mAbs, Afasevikumab targets the IL17A, a proinflammatory cytokine majorly secreted by Th17-cells^39^. Similarly, sequentially distant antibodies, Tremelimumab and Ipilimumab target CTLA4, an immune checkpoint receptor found majorly on T cells^40^. The three-dimensional structures of the Afasevikumab, Ipilimumab and Tremelimumab therapeutic antibodies with their native targets (IL17A and CTLA4 respectively) (PDB codes: 6PPG, 5TRU and 5GGV, respectively) are also available in Protein Data Bank, PDB^25^.

### Comprehensive analysis of the selected antibody pairs

Among the three therapeutic antibodies mentioned in the previous section, Tremelimumab paired with two SARS-CoV-2 specific neutralizing antibodies, C002 and COVA2-29 (Table 2). These neutralizing antibodies had sequence identity of 93.5% with each other (Table S3b). The sequence alignment for the selected antibody pairs is given in Figure S2. The structural similarity between these selected antibody pairs was assessed by superimposing the modelled/crystal structures of respective antibody pairs (neutralizing and therapeutic antibodies) and calculating the root mean square deviation (RMSD) for the aligned regions (Figure S3). Overall, Tremelimumab had the least RMSD (0.44 Å with C002 and 0.35 Å with COVA2-29) over the aligned segments. Afasevikumab and Ipilimumab showed a RMSD of 0.59 Å and 0.92 Å with their neutralizing antibody counterpart, COV2-2015 and HbnC3t1p1_G4, respectively.

**Table 2:** Final list of screened SARS-CoV-2 specific neutralizing and therapeutic antibodies.

Neutralizing antibody		Therapeutic antibody		Sequence identity (%)
Name	Length (aa)	Name	Length (aa)	
C002	231	Tremelimumab	232	90
COV2-2015	230	Afasevikumab	231	92
COVA2-29	232	Tremelimumab	232	90
HbnC3t1p1_G4	224	Ipilimumab	226	90

### Identification of epitopes on SARS-CoV-2 spike protein for docking with antibodies

A “known epitope dataset” was constructed using the information deduced from crystal structures of eight SARS-CoV-2 neutralizing antibodies and epitope information available in the literature (Table S4). These data were used to identify the most optimal antibody-spike protein binding conformation obtained from the docking studies. We have also included epitopes predicted by EpiPred^34^ and the ACE2 binding site on spike protein to examine the overlap of binding site of the selected conformation with in silico predicted epitopes and potential competition with ACE2 binding site. The structure-based epitope prediction method EpiPred predicted the same region on the spike protein as an epitope for all neutralizing and therapeutic antibodies (Table S4).

### Overlapping epitope regions, interaction energy and interface area of neutralizing and therapeutic antibodies

**Figure 2:**
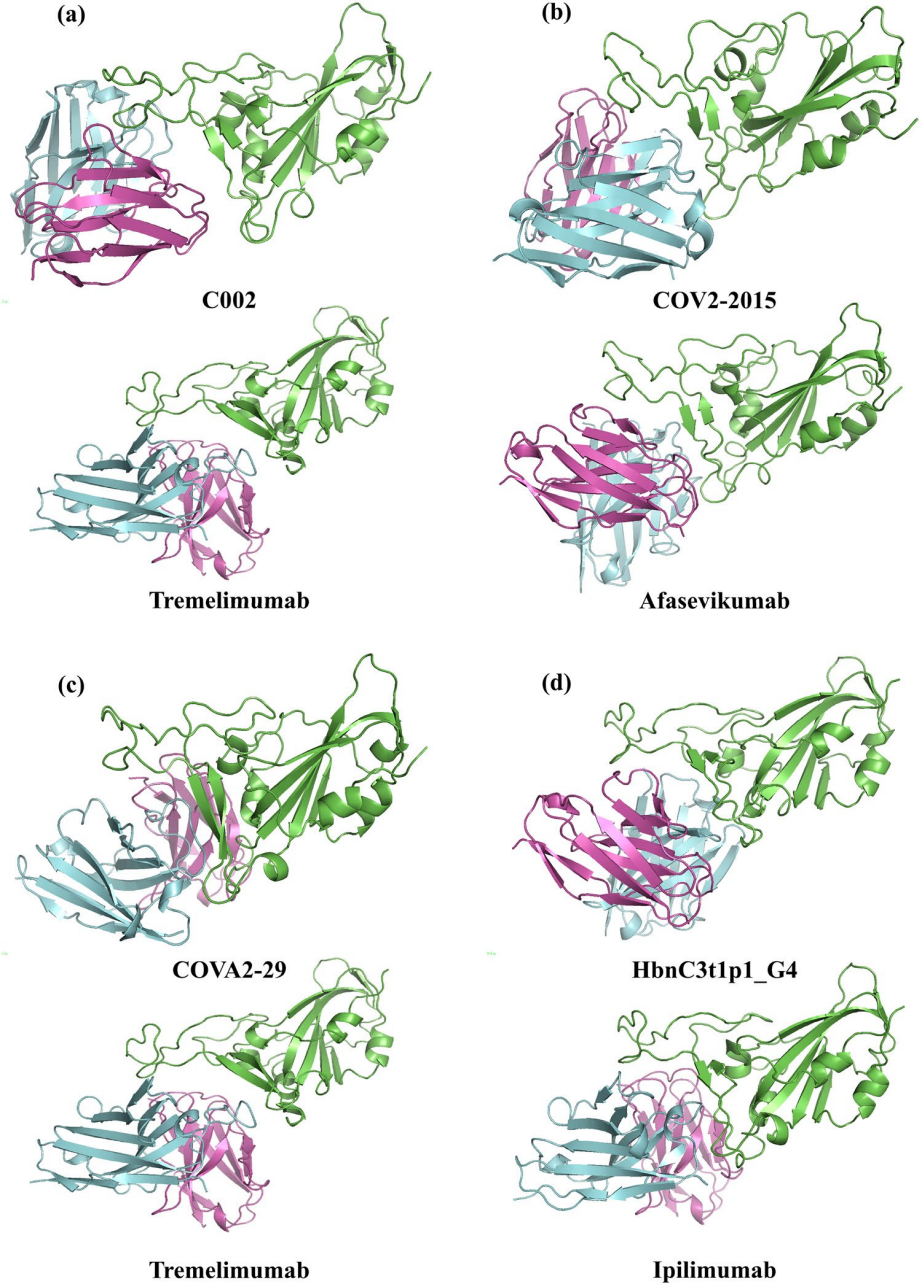
Docked conformation of the SARS-CoV-2 neutralizing antibody (top) and therapeutic antibody (bottom) (light chain: violet; heavy chain: cyan) with spike protein (green) for the pairs (**a**) C002 and Tremelimumab (**b**) COV2-2015 and Afasevikumab (**c**) COVA2-29 and Tremelimumab, and (**d**) HbnC3t1p1_G4 and Ipilimumab. The figures are generated using PyMOL 2.4 (https://pymol.org/2/).

The docked conformations of each antibody with spike protein were assessed based on interaction energy and overlap with the epitopes in “known epitope dataset” (Table 3). The interaction area and length of the epitope were considered in addition, where the above criteria were inadequate to select the optimum conformation. The selected complex structures of neutralizing/therapeutic antibodies with spike protein are shown in Fig. 2 and the details on overlapping residues with known epitopes, interaction energy and interaction area are given in Table 3.

**Table 3:** Summary of epitope regions, interaction energy and interface area for selected conformation of neutralizing and therapeutic antibodies bound with SARS-CoV-2 spike protein.

Antibody	Epitope region	Length	Overlapping residues with known epitopes					Interaction energy (kcal/mol)	Interaction area (Å^2^)
			EPIPRED (29 residues)	7C01 (26 residues)	S2H14 (23 residues)	S2H13 (20 residues)	ACE2 (17 residues)		
**C002**	417,449,453,455,456,473,475,484,485,486,487,488,489,490,493,494,496,498,501,505	20	11	11	**14**	10	14	− 17.2	855
**Tremelimumab**	403,406,417,449,453,455,456,484,485,486,487,488,489,493,494,495,496,498,500,501,502,505	22	8	12	**17**	9	15	− 15.9	848
**COV2-2015**	346,444,445,446,447,448,449,450,452,453,484,485,486,490,493,494,496,498,505	19	4	3	**12**	**12**	8	− 12.7	827
**Afasevikumab**	403,444,445,446,447,449,450,453,455,483,484,485,486,490,493,494,496,498,505	19	6	5	**14**	**13**	9	− 14.9	923
**COVA2-29**	403,405,406,408,409,416,417,446,447,449,453,455,493,494,496,498,500,501,502,503,504,505	22	2	12	**14**	6	12	− 15.6	934
**Tremelimumab**	403,406,417,449,453,455,456,484,485,486,487,488,489,493,494,495,496,498,500,501,502,505	22	8	12	**17**	9	15	− 15.9	848
**HbnC3t1p1_G4**	403,405,408,409,416,417,420,421,449,453,455,456,475,484,486,487,489,490,493,494,496,498,500,501,502,504,505	27	10	**18**	**16**	8	16	− 19.9	955
**Ipilimumab**	403,406,417,421,444,446,447,449,453,455,456,484,485,486,487,489,493,496,498,500,501,502,505	23	9	**13**	**18**	10	16	− 13.7	1039

Epitopes for neutralizing and therapeutic antibodies, which show the highest overlap with known epitope regions are highlighted.

Very interestingly, our results revealed that SARS-CoV-2 neutralizing antibodies and their corresponding therapeutic antibodies exibhit the highest binding overlap with the same epitope regions on SARS-CoV-2 spike protein (Table 3). Tremelimumab and Afasevikumab showed similar interaction energies and overlap with the known epitopes. Afasevikumab and corresponding neutralizing antibody COV2-2015 showed almost equal overlap with S2H14 and S2H13 epitopes. However, Tremelimumab and corresponding neutralizing antibodies C002 and COVA2-29 exclusively bind to S2H14 epitope. The interaction energy of the Tremelimumab was 1.3 kcal/mol weaker than the C002 and 0.3 kcal/mol stronger than the COVA2-29. Ipilimumab showed poor binding to the spike protein than its neutralizing antibody counterpart with an interaction energy difference of 6.2 kcal/mol. On the other hand, Afasevikumab showed better binding than the corresponding neutralizing antibody with an increase of 2.2 kcal/mol in interaction energy. Tremelimumab exhibited marginally stronger binding affinity with spike protein (− 15.9 kcal/mol) than COVA2-29 (− 15.6 kcal/mol) but weaker than C002 (− 17.2 kcal/mol). We observed that the interaction energies of the therapeutic antibodies, tremelimumab, Ipilimumab and afasevikumab with their cognate receptors are − 24.70 kcal/mol, − 18.13 kcal/mol and − 14.23 kcal/mol, respectively. Further, afasevikumab is the only therapeutic antibody with better binding to spike protein (− 14.9 kcal/mol) than its cognate receptor (− 14.23 kcal/mol).

### Analysis of common contacts for therapeutic antibodies

The contacting amino acid residues of the therapeutic antibodies were compared to the contacts on the spike protein and their cognate native target (Table 4). The amino acid residues on antibody (paratope) interacting with both epitopes of spike protein and cognate target were 53.8% (14 out of 26 residues) for Afasevikumab, 59.1% (13 out of 22 residues) for Ipilimumab and 68.2% (15 out of 22 residues) for Tremelimumab. The identical contacts on the spike protein and cognate receptor were further identified for each paratope residue. Our results showed that Afasevikumab harbored the least number of paratope residues with common contacts (2 out of 14 contacts; 14.3%) in the respective epitope, followed by Ipilimumab (5 out of 13 contacts; 38.5%). Tremelimumab had the highest number of paratope residues with common contacts in respective epitopes (9 out of 15; 60%). Overall, Tremelimumab had the highest number of common paratope and epitope residues for spike protein and the cognate receptor CTLA4. The contacts on epitopes, forming multiple contacts with paratope residues of Tremelimumab, include E498Q in spike and Y41Q/Y45Q in CTLA4 in contact with paratope residues H105L, H106Y and H107Y; and E489Y in spike and Y104Y in CTLA4 forming contacts with paratope residues H59Y, L93S and L94T. Such residues, forming multiple contacts with the paratope of antibody, are potentially important for the recognition and effective binding of the antibody to the target.

**Table 4:**
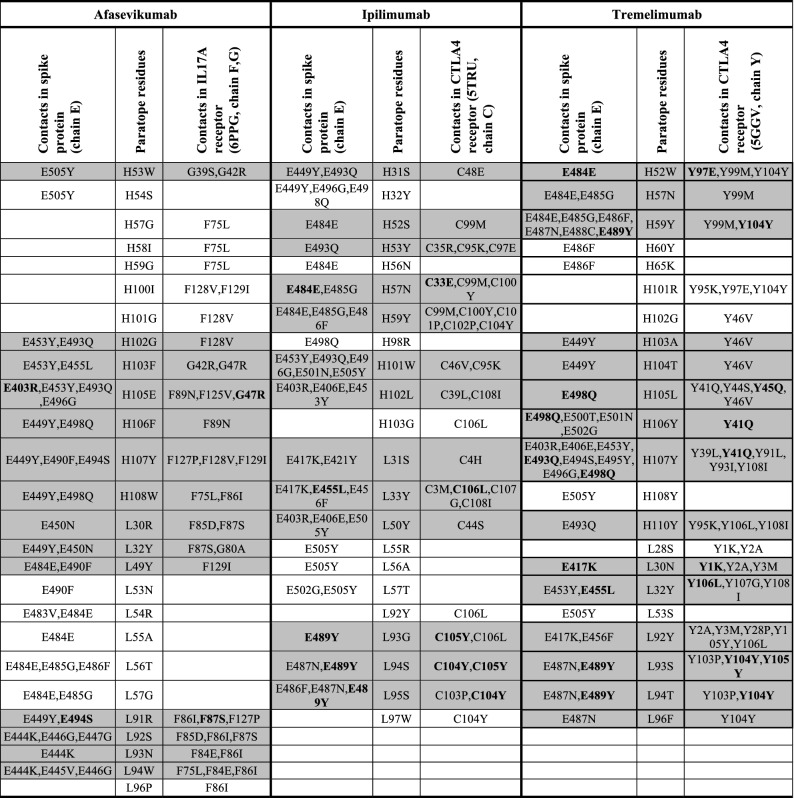
Comparison of the contacting amino acid residues of therapeutic antibodies with SARS-CoV-2 spike protein and their respective native target.

First letter indicates the polypeptide chain followed by amino acid residue number and notation. Highlighted amino acid residues show the common contacts in spike protein and respective native target.

## Discussion

In this work, we have assessed the antibodies, either approved and/or in clinical trials, for their potential to mimic SARS-CoV-2 neutralizing antibodies. The therapeutic antibodies, sequentially similar to neutralizing antibodies, were screened on the basis of length of CDRH3 region and biological significance of their ability to bind to the known epitopes on the RBD region of spike protein. The candidate antibodies Afasevikumab, Ipilimumab, and Tremelimumab, were thus shortlisted and further scrutinized. Interestingly, sequentially distant candidate therapeutic antibodies Ipilimumab and Tremelimumab have the same native target, CTLA4. Moreover, these selected candidate therapeutic antibodies paired with different neutralizing antibodies (Table 2), yet showed maximum binding overlap with the same S2H14 epitope (Table 3). CTLA4 is an inhibitory co-receptor that has been scored as one of the exhaustion markers on T cells and whose levels have been assessed in COVID-19 patients^41^. Severe COVID-19 has been shown to be associated with sustained multifaceted cellular immunosuppression. CTLA4 and PD-1 levels have been shown to be enhanced, with CD4 T cells and Treg cells transiently overexpressing CTLA4 in first few days of post-infection^42–44^. Also, SARS-CoV-2–specific CD4 + T cells from intensive care unit (ICU) patients had significantly higher expression of CTLA-4 than CD4 T cells from convalescent individuals^45^. Further, CTLA-4, has been proposed as a candidate molecule with potential for controlling inflammation in severe COVID-19 patients in the context of Treg based disease management in COVID-19^46^.

On the other hand, cytokine storm is a hallmark of acute respiratory distress syndrome (ARDS) of SARS-CoV-2 infection. IL17, a native target of Afasevikumab, being a pro-inflammatory cytokine, has been considered as a target for drug development for COVID-19^47,48^. Also, given that IL17 is upstream of IL6, an established marker of severity in COVID-19^49,50^, it is noteworthy that an anti-IL17 antibody shares resemblance with a SARS-CoV-2 neutralizing antibody.

As for the binding, antibodies are unique and differ in several ways compared to chemical compounds. Their interaction with antigen is not majorly dependent on the structural complementarity alone. In fact, their interacting surfaces are generally flat and the interactiveness relies mainly on the atomic interactions^51^. They also bind to specific regions on the antigens called epitopes. Therefore, to effectively identify biologically relevant antibodies for the present context, we sampled all possible docked conformations of the shortlisted therapeutic/neutralizing antibodies on the spike protein and selected the best conformation based on the overlap with regions considered as epitopes on the RBD of the spike protein as well as the interaction energy of the whole complex. Among the selected candidate antibodies, Tremelimumab exhibited a good binding to the SARS-CoV-2 spike protein (− 15.9 kcal/mol) comparable to the neutralizing antibodies C002 (− 17.2 kcal/mol) and COVA2-29 (− 15.6 kcal/mol). Tremelimumab also showed similar contacting paratope-epitope residue pairs for spike protein and native cognate receptor CTLA4. Further, Afasevikumab showed better binding energy than its corresponding neutralizing antibody (COV2-2015). However, it exhibited unique paratope-epitope contacts in spike protein and its native receptor.

Antibodies based therapeutics would be required to tackle COVID-19 even after the availability of the vaccines, mainly for treating elderly and people with compromised immune system. Based on our results, we put forward the following contexts of how these antibodies can be applied in COVID-19 management: (1) for lone activity of plausible virus neutralization or immunomodulation; (2) for dual activity exhibiting both neutralization and immunomodulation. This will be better applicable in conditions where both of these activities are preferred, for example, in case of SARS-CoV-2 sepsis^52,53^, where both viral persistence and exacerbated inflammation are present. We strongly opine this will be a major ground for application of these candidate antibodies. (3) For cocktails of anti-immune checkpoint antibodies and anti-cytokine antibodies; (4) for possible inclusion in diagnostic kits for targeted diagnostics in specific suspect groups after careful standardization.

It is of course discernible that caution must be exercised before exploring these candidate antibodies in clinic. Ipilimumab and Tremelimumab have known adverse side effects such as skin reaction, diarrhoea, nausea, fatigue etc.^54,55^ when used for cancer conditions and we believe though such adverse effects cannot be directly anticipated/extrapolated for the present situation, further scrutiny is required before the on field applications. Afasevikumab has only completed phase one clinical trials and its side effects are not known^56^. Experimental evidences are also required for proving their neutralization capacity. Also, before using them for specific purposes owing to their dual activity (as anti-viral as well as immune modulators), crucial factors such as time point post-infection (as the initial stages of infection require anti-viral activity *vis-a-vis,* later stages require immuno-modulatory activity), dose, temporal windows for administration, turnover rate of the antibodies in vivo and need for repeat injections require to be carefully gauged out.

The concept of in silico antibody design has been around for quite some time now^57,58^. However, computational resources developed recently for such analyses have led to very few studies mostly related to optimization of already available antibodies^59,60^. In the present work, although experimental validation is not reported, results from the computational analyses stem from employing experimentally derived datasets: (1) neutralizing antibodies are experimentally verified to neutralize the SARS-CoV-2 and most of these sequences are derived from patients; (2) therapeutic antibodies and their cognate receptors are experimentally evaluated. The interacting residues of the therapeutic antibody-cognate receptor complex are taken from the experimentally determined PDB structures; (3) The epitope (on the RBD region of Spike protein of SARS-CoV-2) dataset used in the study is taken from the experimentally determined PDB structures of spike protein-neutralizing antibody complexes and from literature involving peptide microarray experiments to map linear epitopes on spike protein of SARS-Cov-2 using sera from convalescent COVID-19 patients and (4) the immunosuppression activity is experimentally established for the therapeutic antibodies. All of these in turn add a level of credibility to the observations. Taken together, we have presented a novel computational pipeline which will have significantly high potential in the near future when a large pool of therapeutic antibodies will be available for potential repurposing of therapeutic antibodies and hence might find broader applications in the intervention of other similar clinical conditions.

## Supplementary Information

Supplementary Information 1.

## Abbreviations

RBD: Receptor-binding domain of spike protein
ACE2: Angiotensin-converting enzyme 2
mAbs: Monoclonal antibody
V_H_ and V_L_: Variable region of heavy and light chains of antibody
CDRH3 andCDRL3: Complementarity-determining regions of heavy and light chain of antibody
RMSD: Root mean square deviation
aa: Amino acid
