# Supplementary material for: Exploring antibody repurposing for COVID-19: beyond presumed roles of therapeutic antibodies
Source: Sci Rep. 2021 May 13;11:10220. doi: 10.1038/s41598-021-89621-6 (PMC8119408; doi:10.1038/s41598-021-89621-6)
Supplement: Supplementary file 1 — Supplementary Information 1. [file 41598_2021_89621_MOESM1_ESM.docx]

**Exploring antibody repurposing for COVID-19: Beyond presumed roles of therapeutic antibodies**

Puneet Rawat^1^, Divya Sharma^1^, Ambuj Srivastava^1^, Vani Janakiraman^2,*^, M Michael Gromiha^1,*^

# ^1^Protein Bioinformatics Lab, Department of Biotechnology, Bhupat and Jyoti Mehta School of Biosciences, Indian Institute of Technology Madras, Chennai – 600036, Tamil Nadu, India.

# ^2^Infection Biology Lab, Department of Biotechnology, Bhupat and Jyoti Mehta School of Biosciences, Indian Institute of Technology Madras, Chennai – 600036, Tamil Nadu, India.

**Keywords:** COVID-19, Neutralizing antibodies, Antibody repurposing, SARS-CoV-2.

*Corresponding authors

MMG: [gromiha@iitm.ac.in](mailto:gromiha@iitm.ac.in)

VJ: [vani@iitm.ac.in](mailto:vani@iitm.ac.in)

**Supplementary information**

**Table S1.** List of selected SARS-CoV-2 neutralizing (rows) and therapeutic (columns) antibody pairs at 90% identity cutoff. Candidate antibody pairs selected after primary screening are highlighted in bold font.

| Antibodies |  | **Afasevikumab** | Atoltivimab | Avdoralimab | Avelumab | **Balstilimab** | Berlimatoxumab | **Bifikafusp** | Bimagrumab | Bintrafusp | **Camidanlumab** | Carlumab | Cetrelimab | **Cudarolimab** | **Daratumumab** | **Denosumab** | Durvalumab | **Elgemtumab** | **Enapotamab** | Evolocumab | Foralumab | Foravirumab | **Ipilimumab** | Lexatumumab | Lirilumab | **Maftivimab** | **Marstacimab** | Nivolumab | **Nurulimab** | **Ofatumumab** | Onvatilimab | **Opicinumab** | Pacmilimab | Sifalimumab | **Simlukafusp** | Sugemalimab | **Tarextumab** | **Tremelimumab** | Varlilumab |
| --- | --- | --- | --- | --- | --- | --- | --- | --- | --- | --- | --- | --- | --- | --- | --- | --- | --- | --- | --- | --- | --- | --- | --- | --- | --- | --- | --- | --- | --- | --- | --- | --- | --- | --- | --- | --- | --- | --- | --- |
|  | Length (aa) | **231** | 224 | 231 | 230 | **220** | 226 | **224** | 226 | 230 | **222** | 228 | 230 | **230** | **229** | **230** | 229 | **224** | **224** | 224 | 226 | 226 | **226** | 229 | 230 | **224** | **232** | 220 | **226** | **229** | 227 | **226** | 223 | 224 | **225** | 229 | **223** | **232** | 226 |
| **C002** | **231** |  | 91 |  |  |  |  |  |  |  |  |  |  |  |  |  |  |  |  |  |  | 91 |  |  |  | 90 |  |  |  |  |  |  | 90 |  |  |  |  | **90** | 92 |
| **C003** | **225** |  |  | 92 |  |  |  | **91** |  |  |  |  |  |  |  | 92 |  |  | **92** |  |  |  |  |  |  |  |  |  |  |  |  |  |  |  |  |  |  |  |  |
| C013 | 245 |  |  |  |  |  |  |  |  |  | 90 | 92 | 95 |  |  |  |  |  |  |  |  |  |  |  | 93 |  |  |  |  |  |  |  |  |  |  |  |  |  |  |
| **C017** | **229** |  |  |  |  |  |  |  |  |  |  |  |  |  |  |  |  |  |  |  |  |  |  |  |  |  |  |  |  | **92** |  |  |  |  |  |  |  |  |  |
| **C102** | **225** |  |  |  |  |  |  | **90** |  |  |  |  |  |  |  | 92 |  |  | **91** |  |  |  |  |  |  |  |  |  |  |  |  |  |  |  |  |  |  |  |  |
| C121 | 239 |  |  |  |  |  |  |  | 91 |  |  |  |  |  |  |  |  |  |  |  |  |  |  |  |  |  |  |  |  |  |  |  |  |  |  |  |  |  |  |
| C144 | 241 |  |  |  | 91 |  |  |  |  | 91 |  |  |  |  |  |  |  |  |  |  |  |  |  |  |  |  |  |  |  |  |  |  |  |  |  |  |  |  |  |
| C145 | 241 |  |  |  | 91 |  |  |  |  | 91 |  |  |  |  |  |  |  |  |  |  |  |  |  |  |  |  |  |  |  |  |  |  |  |  |  |  |  |  |  |
| **C154** | **234** |  |  |  |  |  |  |  |  |  |  |  |  | **92** |  |  |  |  |  |  |  |  |  |  |  |  |  |  |  |  |  |  |  |  |  |  |  |  |  |
| **C155** | **224** |  |  |  |  | **92** |  |  |  |  |  |  |  |  |  |  |  |  |  |  |  |  |  |  |  |  |  |  |  |  |  |  |  |  |  |  |  |  |  |
| C165 | 230 |  |  |  |  |  |  |  |  |  | 95 |  |  |  |  |  |  |  |  |  |  |  |  |  |  |  |  |  |  |  |  |  |  |  |  |  |  |  |  |
| **C207** | **228** |  |  |  |  |  |  | **91** |  |  |  |  |  |  | **94** | **90** |  |  | **91** |  |  |  |  |  |  |  |  | 90 |  |  |  | **90** |  |  | **91** |  | 91 |  |  |
| **C211** | **224** |  |  |  |  | **92** |  |  |  |  |  |  |  |  |  |  |  |  |  |  |  |  |  |  |  |  |  |  |  |  |  |  |  |  |  |  |  |  |  |
| **CB6** | **228** |  |  |  |  |  |  |  |  |  |  |  |  |  |  |  |  |  |  |  |  |  |  |  |  | **91** |  |  |  |  |  |  |  |  |  |  |  |  |  |
| CC12.19 | 235 |  |  |  |  |  |  |  |  |  |  |  |  |  |  |  |  |  |  |  |  |  |  |  |  |  |  |  |  |  |  |  |  |  |  | 93 |  |  |  |
| **CC12.2** | **225** |  |  |  |  |  |  |  |  |  |  |  |  |  |  | 90 |  |  | **91** |  |  |  |  |  |  |  |  |  |  |  |  |  |  |  |  |  |  |  |  |
| **CC12.3** | **224** |  |  |  |  |  |  |  |  |  |  |  |  |  |  | 91 |  |  | **90** |  |  |  |  |  |  |  |  |  |  |  |  |  |  |  |  |  |  |  |  |
| CC6.30 | 232 |  |  |  |  |  |  |  |  |  |  |  |  |  |  |  |  |  |  |  |  |  |  |  |  |  |  |  |  |  | 91 |  |  |  |  |  |  |  |  |
| **CC6.33** | **226** |  |  |  |  |  |  |  |  |  | **93** |  |  |  |  |  |  |  |  |  |  |  |  |  |  |  |  |  |  |  |  |  |  |  |  |  |  |  |  |
| CnC2t1p1_B4 | 229 |  |  |  |  |  |  |  |  |  |  |  |  |  |  |  |  |  |  | 92 |  |  |  |  |  |  |  |  |  |  |  |  |  |  |  |  |  |  |  |
| **COV2-2015** | **230** | **92** |  |  |  |  |  |  |  |  |  |  |  |  |  |  |  |  |  |  |  |  |  |  |  |  |  |  |  | **92** |  |  |  |  |  |  |  |  |  |
| COV2-2514 | 237 |  |  |  |  |  |  |  |  |  |  |  |  |  |  |  |  |  |  |  |  |  |  | 91 |  |  |  |  |  |  |  |  |  |  |  |  |  |  |  |
| COV2-2678 | 237 |  |  |  |  |  |  |  |  |  |  |  |  |  |  |  |  |  |  |  |  |  |  | 90 |  |  |  |  |  |  |  |  |  |  |  |  |  |  |  |
| COVA2-02 | 231 |  |  |  |  |  | 90 |  |  |  |  |  |  |  |  |  |  |  |  |  |  |  |  |  |  |  |  |  |  |  |  |  |  |  |  |  |  |  |  |
| **COVA2-07** | **223** |  |  |  |  |  |  |  |  |  |  |  |  |  |  | 90 |  |  | **91** |  |  |  |  |  |  |  |  |  |  |  |  |  |  |  |  |  |  |  |  |
| COVA2-11 | 235 |  |  |  |  |  |  |  |  |  | 92 |  |  |  |  |  |  |  |  |  |  |  |  |  |  |  |  |  |  |  |  |  |  |  |  |  |  |  |  |
| **COVA2-13** | **226** |  |  |  |  |  |  |  |  |  |  |  |  |  |  | **90** |  |  | **90** |  |  |  |  |  |  |  |  |  |  |  |  |  |  |  |  |  |  |  |  |
| **COVA2-29** | **232** |  |  |  |  |  |  |  |  |  |  |  |  |  |  |  |  |  |  |  |  | **90** |  |  |  | **91** |  |  |  |  |  |  |  |  |  |  |  | **90** | 91 |
| **CV30** | **226** |  |  |  |  |  |  |  |  |  |  |  |  |  |  | **90** |  |  | **91** |  |  |  |  |  |  |  |  |  |  |  |  |  |  |  |  |  |  |  |  |
| HbnC2t1p2_D9 | 235 |  |  |  |  |  |  |  |  |  |  |  |  |  |  |  |  |  |  |  | 92 |  |  |  |  |  |  | 91 |  |  |  |  |  |  |  |  |  |  |  |
| **HbnC3t1p1_G4** | **224** |  |  |  |  |  |  | **91** |  |  |  |  |  |  |  | 93 | 90 |  | **91** |  |  |  | **90** |  |  |  |  |  | **91** |  |  |  |  |  | **91** |  | **90** |  |  |
| **HbnC3t1p2_B10** | **224** |  |  |  |  |  |  |  |  |  |  |  |  |  |  | 91 |  |  | **90** |  |  |  |  |  |  |  |  |  |  |  |  |  |  |  |  |  |  |  |  |
| **MnC2t1p1_A3** | **226** |  |  |  |  |  |  |  |  |  |  |  |  |  |  |  |  | **91** |  |  |  |  |  |  |  |  |  |  |  |  |  |  |  |  |  |  |  |  |  |
| **MnC2t1p1_C5** | **226** |  |  |  |  |  |  |  |  |  |  |  |  |  |  |  |  | **92** |  |  |  |  |  |  |  | **90** |  |  |  |  |  |  |  |  |  |  |  |  |  |
| REGN10977 | 231 |  |  |  |  |  |  |  |  |  | 93 |  |  |  |  |  |  |  |  |  |  |  |  |  |  |  |  |  |  |  |  |  |  |  |  |  |  |  |  |
| **REGN10986** | **231** |  |  |  |  |  |  |  |  |  |  |  |  |  |  |  |  |  |  |  |  |  |  |  |  |  | **91** |  |  |  |  |  |  |  |  |  |  |  |  |
| S309 | 234 |  |  |  |  |  |  |  |  |  |  |  |  |  |  |  |  |  |  |  |  |  |  |  |  |  |  |  |  |  |  |  |  | 92 |  |  |  |  |  |

aa: amino acid

Redundant therapeutic antibody sequences: (i) Bifikafusp, Onfekafusp and Radretumab: Bifikafusp is used as representative antibody. (ii) Tremelimumab and Ticilimumab: Tremelimumab is used as representative antibody.

**Table S2.** Comparison of complementarity-determining regions of heavy and light chains (CDRH3 and CDRL3) for each SARS-CoV-2 neutralizing antibody-therapeutic antibody pair.

| **SARS-CoV-2 neutralizing antibody** | | | | | **Therapeutic antibody** | | | | |
| --- | --- | --- | --- | --- | --- | --- | --- | --- | --- |
| **Antibodies** | **CDRH3**  **length** | **CDRH3**  **sequence** | **CDRL3**  **length** | **CDRL3**  **sequence** | **Antibodies** | **CDRH3**  **length** | **CDRH3**  **sequence** | **CDRL3**  **length** | **CDRL3**  **sequence** |
| **C002** | **16** | **AKEGRPSDIVVVVAFD** | **8** | **QQSYSTPR** | **Tremelimumab** | **17** | **ARDPRGATLYYYYYGMD** | **8** | **QQYYSTPF** |
| C003 | 10 | ARDYGDFYFD | 8 | QQYGSSPR | Bifikafusp | 8 | AKPFPYFD | 8 | QQTGRIPP |
| C003 | 10 | ARDYGDFYFD | 8 | QQYGSSPR | Enapotamab | 8 | AKIWIAFD | 8 | QQYGSSPY |
| C017 | 18 | AKAGVRGIAAAGPDLNFD | 4 | QQRI | Ofatumumab | 14 | AKDIQYGNYYYGMD | 8 | QQRSNWPI |
| C102 | 10 | ARDYGDYYFD | 8 | QQYGSSPR | Bifikafusp | 8 | AKPFPYFD | 8 | QQTGRIPP |
| C102 | 10 | ARDYGDYYFD | 8 | QQYGSSPR | Enapotamab | 8 | AKIWIAFD | 8 | QQYGSSPY |
| C154 | 19 | AKQAGPYCSGGSCYSAPFD | 8 | QQYDNLPI | Cudarolimab | 16 | ARGRPWYSETGTSAFD | 7 | QQSDHYP |
| C155 | 10 | ARDFGEFYFD | 8 | QQYNNWPR | Balstilimab | 5 | ASNGD | 8 | QQYNNWPR |
| C207 | 13 | AKEPIGQPLLWWD | 8 | QQRSNWPR | Bifikafusp | 8 | AKPFPYFD | 8 | QQTGRIPP |
| **C207** | **13** | **AKEPIGQPLLWWD** | **8** | **QQRSNWPR** | **Daratumumab** | **14** | **AKDKILWFGEPVFD** | **8** | **QQRSNWPP** |
| **C207** | **13** | **AKEPIGQPLLWWD** | **8** | **QQRSNWPR** | **Denosumab** | **14** | **AKDPGTTVIMSWFD** | **8** | **QQYGSSPR** |
| C207 | 13 | AKEPIGQPLLWWD | 8 | QQRSNWPR | Enapotamab | 8 | AKIWIAFD | 8 | QQYGSSPY |
| C207 | 13 | AKEPIGQPLLWWD | 8 | QQRSNWPR | Opicinumab | 10 | ATEGDNDAFD | 9 | QQRSNWPMY |
| C207 | 13 | AKEPIGQPLLWWD | 8 | QQRSNWPR | Simlukafusp | 9 | AKGWFGGFN | 8 | QQGIMLPP |
| C211 | 10 | ARDYGDFYFD | 8 | QQYNNWPR | Balstilimab | 5 | ASNGD | 8 | QQYNNWPR |
| CB6 | 12 | ARVLPMYGDYLD | 10 | QQSYSTPPEY | Maftivimab | 10 | AKRGYPHSFD | 7 | QQSYSTL |
| CC12.2 | 10 | ARDYGDLYFD | 8 | QQYGSSPR | Enapotamab | 8 | AKIWIAFD | 8 | QQYGSSPY |
| CC12.3 | 10 | ARDFGDFYFD | 8 | QQYGSSPR | Enapotamab | 8 | AKIWIAFD | 8 | QQYGSSPY |
| CC6.33 | 10 | ALRNQWDLLV | 8 | QHYGSSLW | Camidanlumab | 7 | ARKDWFD | 8 | QQYGSSPL |
| **COV2-2015** | **14** | **AMGPFGELLPYYFD** | **9** | **QQRSNWPPY** | **Afasevikumab** | **15** | **ARDIGGFGEFYWNFG** | **9** | **QQRSNWPPA** |
| **COV2-2015** | **14** | **AMGPFGELLPYYFD** | **9** | **QQRSNWPPY** | **Ofatumumab** | **14** | **AKDIQYGNYYYGMD** | **8** | **QQRSNWPI** |
| **COVA2-07** | **8** | **AREAYGMD** | **8** | **QQYGSSPG** | **Enapotamab** | **8** | **AKIWIAFD** | **8** | **QQYGSSPY** |
| COVA2-13 | 11 | ARDLDTMGGMD | 8 | QQYGSSPG | Denosumab | 14 | AKDPGTTVIMSWFD | 8 | QQYGSSPR |
| COVA2-13 | 11 | ARDLDTMGGMD | 8 | QQYGSSPG | Enapotamab | 8 | AKIWIAFD | 8 | QQYGSSPY |
| **COVA2-29** | **17** | **ASLPVVPAAIGPLPAFD** | **8** | **QQSYSTPR** | **Tremelimumab** | **17** | **ARDPRGATLYYYYYGMD** | **8** | **QQYYSTPF** |
| CV30 | 11 | ARDLDVSGGMD | 8 | QQYGSSPQ | Denosumab | 14 | AKDPGTTVIMSWFD | 8 | QQYGSSPR |
| CV30 | 11 | ARDLDVSGGMD | 8 | QQYGSSPQ | Enapotamab | 8 | AKIWIAFD | 8 | QQYGSSPY |
| HbnC3t1p1_G4 | 10 | ARDFGDFFFD | 8 | QQYGSSPR | Bifikafusp | 8 | AKPFPYFD | 8 | QQTGRIPP |
| HbnC3t1p1_G4 | 10 | ARDFGDFFFD | 8 | QQYGSSPR | Enapotamab | 8 | AKIWIAFD | 8 | QQYGSSPY |
| **HbnC3t1p1_G4** | **10** | **ARDFGDFFFD** | **8** | **QQYGSSPR** | **Ipilimumab** | **10** | **ARTGWLGPFD** | **8** | **QQYGSSPW** |
| **HbnC3t1p1_G4** | **10** | **ARDFGDFFFD** | **8** | **QQYGSSPR** | **Nurulimab** | **10** | **ARTGWLGPFD** | **8** | **QQYGSSPW** |
| **HbnC3t1p1_G4** | **10** | **ARDFGDFFFD** | **8** | **QQYGSSPR** | **Simlukafusp** | **9** | **AKGWFGGFN** | **8** | **QQGIMLPP** |
| HbnC3t1p1_G4 | 10 | ARDFGDFFFD | 8 | QQYGSSPR | Tarextumab | 7 | ARSIFYT | 8 | QQYSNFPI |
| HbnC3t1p2_B10 | 10 | ARDYGDYFFD | 8 | QQYGSSPR | Enapotamab | 8 | AKIWIAFD | 8 | QQYGSSPY |
| MnC2t1p1_A3 | 12 | ATGARFGESPFD | 8 | QQANSFPG | Elgemtumab | 9 | ARWGDEGFD | 8 | QQYSSFPT |
| MnC2t1p1_C5 | 12 | ATGARFGESPFD | 8 | QQANSFPG | Elgemtumab | 9 | ARWGDEGFD | 8 | QQYSSFPT |
| MnC2t1p1_C5 | 12 | ATGARFGESPFD | 8 | QQANSFPG | Maftivimab | 10 | AKRGYPHSFD | 7 | QQSYSTL |
| **REGN10986** | **12** | **ARALPYGDLHFD** | **11** | **QSYDSSLSDSY** | **Marstacimab** | **12** | **AILGATSLSAFD** | **11** | **QSYDSSLSGSG** |

**Table S3.** Percent identity obtained amongst (a) therapeutic and (b) SARS-CoV-2 neutralizing antibodies.

| **(a) Therapeutic antibodies** | | | | | | | | | | |
| --- | --- | --- | --- | --- | --- | --- | --- | --- | --- | --- |
|  | **Afasevikumab** | **Daratumumab** | **Denosumab** | **Enapotamab** | **Ipilimumab** | **Marstacimab** | **Nurulimab** | **Ofatumumab** | **Simlukafusp** | **Tremelimumab** |
| **Afasevikumab** | 100 |  |  |  |  |  |  |  |  |  |
| **Daratumumab** | 89.1 | 100 |  |  |  |  |  |  |  |  |
| **Denosumab** | 83.9 | 88.2 | 100 |  |  |  |  |  |  |  |
| **Enapotamab** | 84.4 | 89.3 | 93.8 | 100 |  |  |  |  |  |  |
| **Ipilimumab** | 85 | 85.8 | 88.9 | 89.3 | 100 |  |  |  |  |  |
| **Marstacimab** | 67.5 | 72.5 | 74.3 | 76.3 | 70.8 | 100 |  |  |  |  |
| **Nurulimab** | 85.4 | 86.3 | 89.4 | 89.7 | **99.6** | 70.8 | 100 |  |  |  |
| **Ofatumumab** | 90.4 | 87.3 | 83 | 86.2 | 84.1 | 67.2 | 84.5 | 100 |  |  |
| **Simlukafusp** | 84.9 | 90.2 | 91.6 | 91.5 | 88.4 | 73.8 | 88.9 | 84 | 100 |  |
| **Tremelimumab** | 74.5 | 75.1 | 76.5 | 76.8 | 81 | 70.7 | 80.5 | 76.9 | 75.6 | 100 |

Nurulimab is a biosimilar of Ipilimumab (99.6%). Hence, Ipilimumab is used as a representative for further analysis to avoid redundancy.

| **(b) SARS-CoV-2 neutralizing antibodies** | | | | | | | |
| --- | --- | --- | --- | --- | --- | --- | --- |
|  | **C002** | **COVA2-29** | **C207** | **COV2-2015** | **COVA2-07** | **HbnC3t1p1_G4** | **REGN10986** |
| **C002** | 100 |  |  |  |  |  |  |
| **COVA2-29** | 93.5 | 100 |  |  |  |  |  |
| **C207** | 80.3 | 80.3 | 100 |  |  |  |  |
| **COV2-2015** | 76.5 | 76.5 | 89 | 100 |  |  |  |
| **COVA2-07** | 78.9 | 79.4 | 87.9 | 83 | 100 |  |  |
| **HbnC3t1p1_G4** | 81.3 | 80.4 | 89.7 | 86.2 | 92.8 | 100 |  |
| **REGN10986** | 71 | 70.6 | 71.5 | 68.3 | 74.4 | 77.2 | 100 |

**Table S4.** Regions proposed as epitopes and ACE2-binding site interface residue positions in the SARS-CoV-2 spike protein.

| **Epitope** | **Length** | **Amino acid residue position** |
| --- | --- | --- |
| **B38 (7BZ5)** | 29 | 403,405,409,415,416,417,420,421,453,455,456,457,458,473,475,476,477,484,486,487,489,490,495,496,498,500,501,502,505 |
| **CB6 (7C01)** | 26 | 403,405,406,408,409,415,416,417,420,421,455,456,457,458,473,474,475,476,477,486,487,489,493,501,502,505 |
| **P2B-2F6 (7BWJ)** | 13 | 346,444,446,447,448,449,450,452,483,484,485,490,494 |
| **CV30 (6XE1)** | 23 | 403,415,416,417,420,421,453,455,456,457,458,460,473,475,476,477,486,487,489,493,501,502,505 |
| **COVA2-39 (7JMP)** | 11 | 446,449,456,475,483,484,485,486,487,489,493 |
| **COVA2-04 (7JMO)** | 28 | 403,405,415,416,417,420,421,453,455,456,457,458,460,473,475,476,477,486,487,489,493,494,495,496,500,501,502,505 |
| **CC12.1 (6XC2)** | 32 | 403,405,408,415,416,417,420,421,453,455,456,457,458,459,460,473,474,475,476,477,486,487,489,493,494,495,496,498,500,501,502,505 |
| **CC12.3 (6XC4)** | 23 | 403,405,415,416,417,420,421,453,455,456,457,458,460,473,475,476,477,486,487,489,495,501,505 |
| **S2H14^$^** | 23 | 403,444,445,446,447,449,453,455,456,475,485,487,489,493,494,495,496,498,499,500,501,502,505 |
| **S2H13^$^** | 20 | 444,445,446,447,449,472,479,480,481,482,483,484,485,486,488,489,490,493,494,498 |
| **Linear epitope^&^** | 12 | 487,488,489,490,491,492,493,494,495,496,497,498 |
| **ACE2** | 17 | 417,446,449,453,455,456,475,486,487,489,493,496,498,500,501,502,505 |
| **EpiPred^*^** | 29 | 417,421,455,456,457,458,459,460,465,467,469,470,471,472,473,474,475,476,477,478,479,482,483,484,485,486,487,489,490 |

^$^ Epitopes mapped on the spike protein for neutralizing antibodies from Piccoli et al., [2020]

^&^ Linear epitope on the spike protein from Li et al., [2020]

^*^ Computationally predicted epitopes using EpiPred for all neutralizing and therapeutic antibodies.

Interestingly, same epitope residues were predicted for all antibodies.

**Table S5.** Sequence identity (%) amongst different regions considered as epitopes from SARS-CoV-2 spike protein.

|  |  | **7BZ5 (B38)** | **CB6 (7C01)** | **P2B-2F6 (7BWJ)** | **CV30 (6XE1)** | **COVA2-39 (7JMP)** | **COVA2-04 (7JMO)** | **CC12.1 (6XC2)** | **CC12.3 (6XC4)** | **S2H14^$^** | **S2H13^$^** | **Linear epitope^#^** | **ACE2** | **EpiPred** |
| --- | --- | --- | --- | --- | --- | --- | --- | --- | --- | --- | --- | --- | --- | --- |
|  | **Length** | 29 | 26 | 13 | 23 | 11 | 28 | 32 | 23 | 23 | 20 | 12 | 17 | 29 |
| **B38 (7BZ5)** | 29 | 100 | 76 | 7 | 72 | 21 | 86 | 90 | 76 | 48 | 17 | 21 | 48 | 52 |
| **CB6 (7C01)** | 26 | 85 | 100 | 0 | 81 | 23 | 85 | 92 | 77 | 38 | 12 | 12 | 42 | 54 |
| **P2B-2F6 (7BWJ)** | 13 | 15 | 0 | 100 | 0 | 38 | 8 | 8 | 0 | 46 | 69 | 15 | 15 | 31 |
| **CV30 (6XE1)** | 23 | 91 | 91 | 0 | 100 | 26 | 100 | 100 | 91 | 48 | 13 | 13 | 52 | 61 |
| **COVA2-39 (7JMP)** | 11 | 55 | 55 | 45 | 55 | 100 | 55 | 55 | 45 | 73 | 73 | 27 | 73 | 73 |
| **COVA2-04 (7JMO)** | 28 | 89 | 79 | 4 | 82 | 21 | 100 | 100 | 82 | 54 | 14 | 21 | 50 | 50 |
| **CC12.1 (6XC2)** | 32 | 81 | 75 | 3 | 72 | 19 | 88 | 100 | 72 | 50 | 16 | 22 | 47 | 50 |
| **CC12.3 (6XC4)** | 23 | 96 | 87 | 0 | 91 | 22 | 100 | 100 | 100 | 43 | 9 | 13 | 43 | 61 |
| **S2H14^$^** | 23 | 61 | 43 | 26 | 48 | 35 | 65 | 70 | 43 | 100 | 43 | 30 | 65 | 26 |
| **S2H13^$^** | 20 | 25 | 15 | 45 | 15 | 40 | 20 | 25 | 10 | 50 | 100 | 30 | 30 | 45 |
| **Linear epitope^&^** | 12 | 50 | 25 | 17 | 25 | 25 | 50 | 58 | 25 | 58 | 50 | 100 | 42 | 25 |
| **ACE2** | 17 | 82 | 65 | 12 | 71 | 47 | 82 | 88 | 59 | 88 | 35 | 29 | 100 | 41 |
| **EpiPred** | 29 | 52 | 48 | 14 | 48 | 28 | 48 | 55 | 48 | 21 | 31 | 10 | 24 | 100 |

(i) Percent identity = Number of identical residues*100/length of the epitope (row).

(ii) Epitopes highlighted are the unique ones that comply with the average epitope length (~22 amino acid residues)

(iii) Epitopes not overlapping with the ACE2 binding site were not considered for the study.

^$^ Epitopes mapped on the spike protein for neutralizing antibodies from Piccoli et al., [2020]

^&^ Linear epitope on the spike protein from Li et al., [2020]

**Figure S1.** Total number of antibody pairs and number of unique SARS-CoV-2 neutralizing/therapeutic antibodies selected at different sequence identity cutoffs.


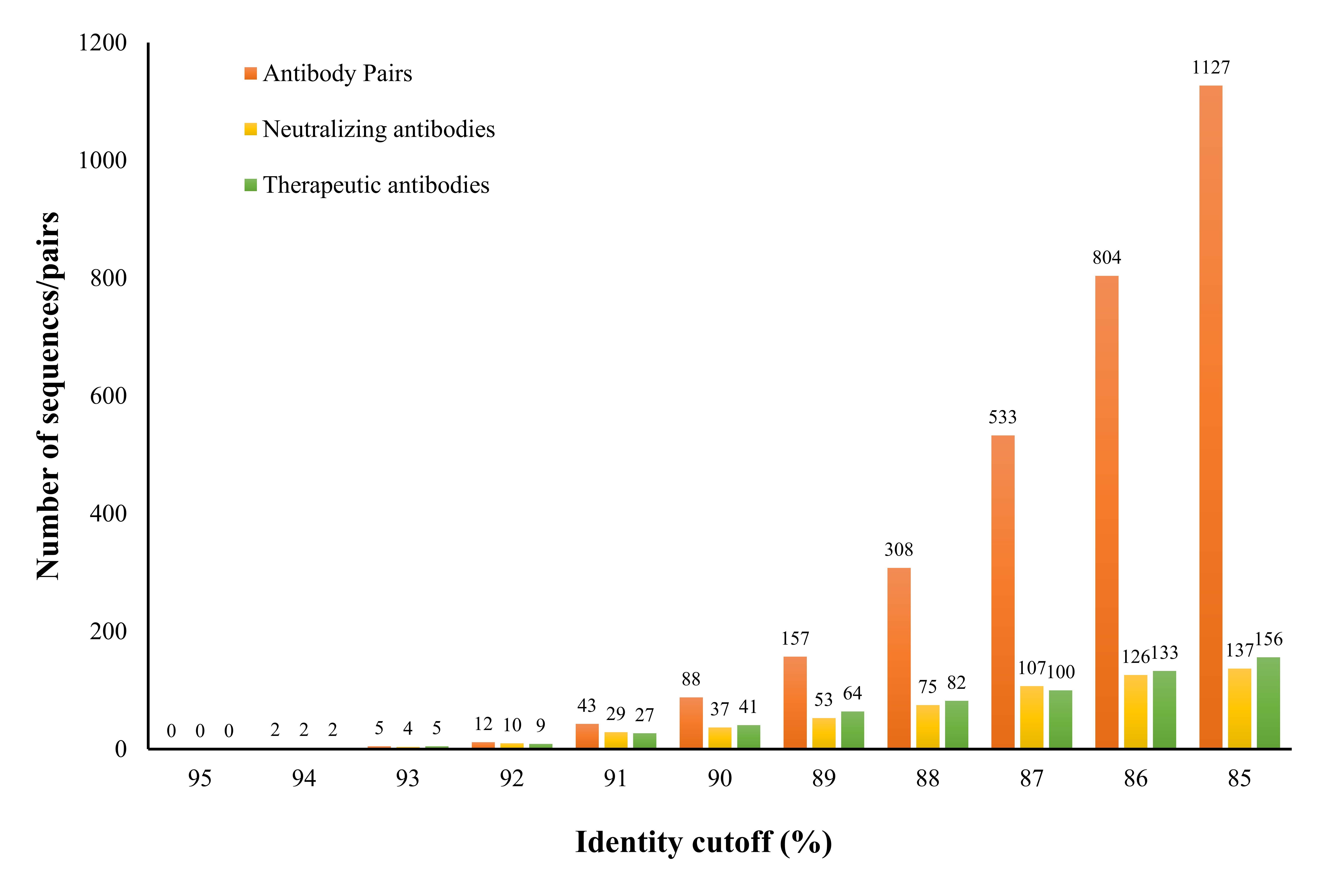


**Figure S2.** Sequence alignment of the shortlisted SARS-CoV-2 neutralizing antibody and corresponding therapeutic antibody pairs.


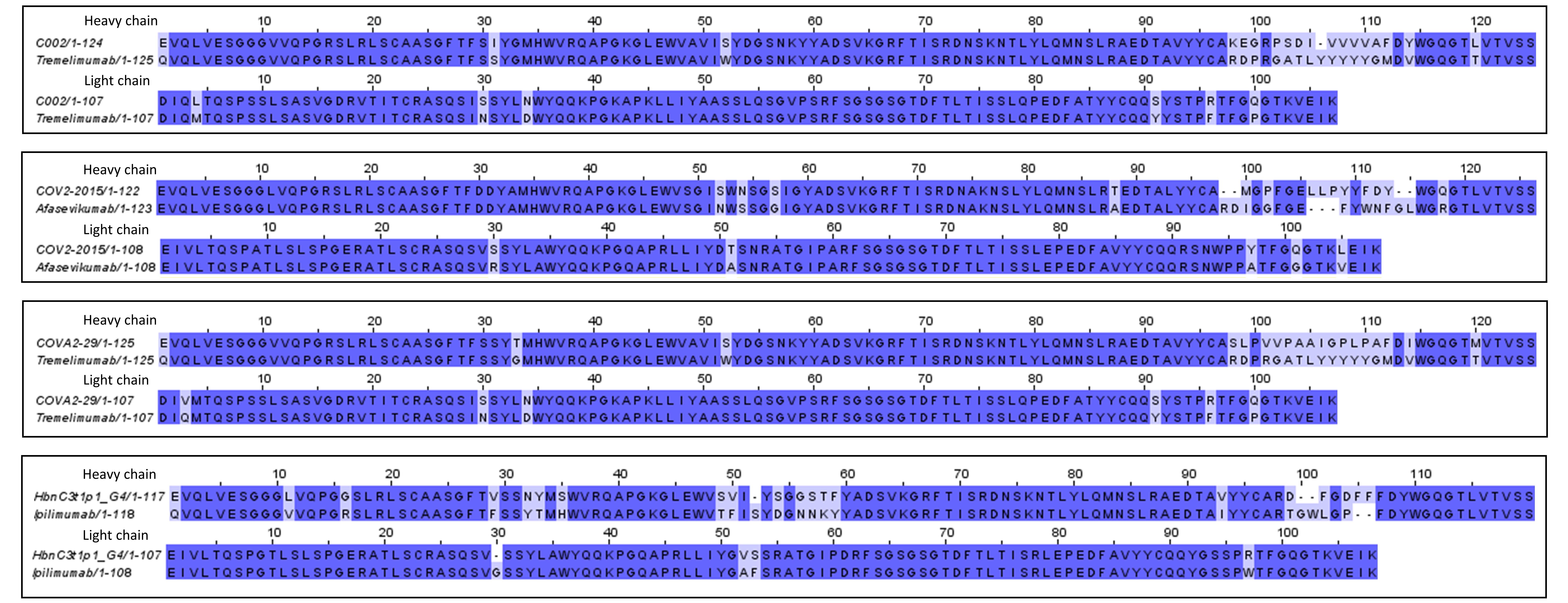


**Figure S3.** Superimposed structures of the selected SARS-CoV-2 neutralizing antibodies (green) and sequentially similar therapeutic antibodies (blue). The figures are generated using PyMOL 2.4 (https://pymol.org/2/).


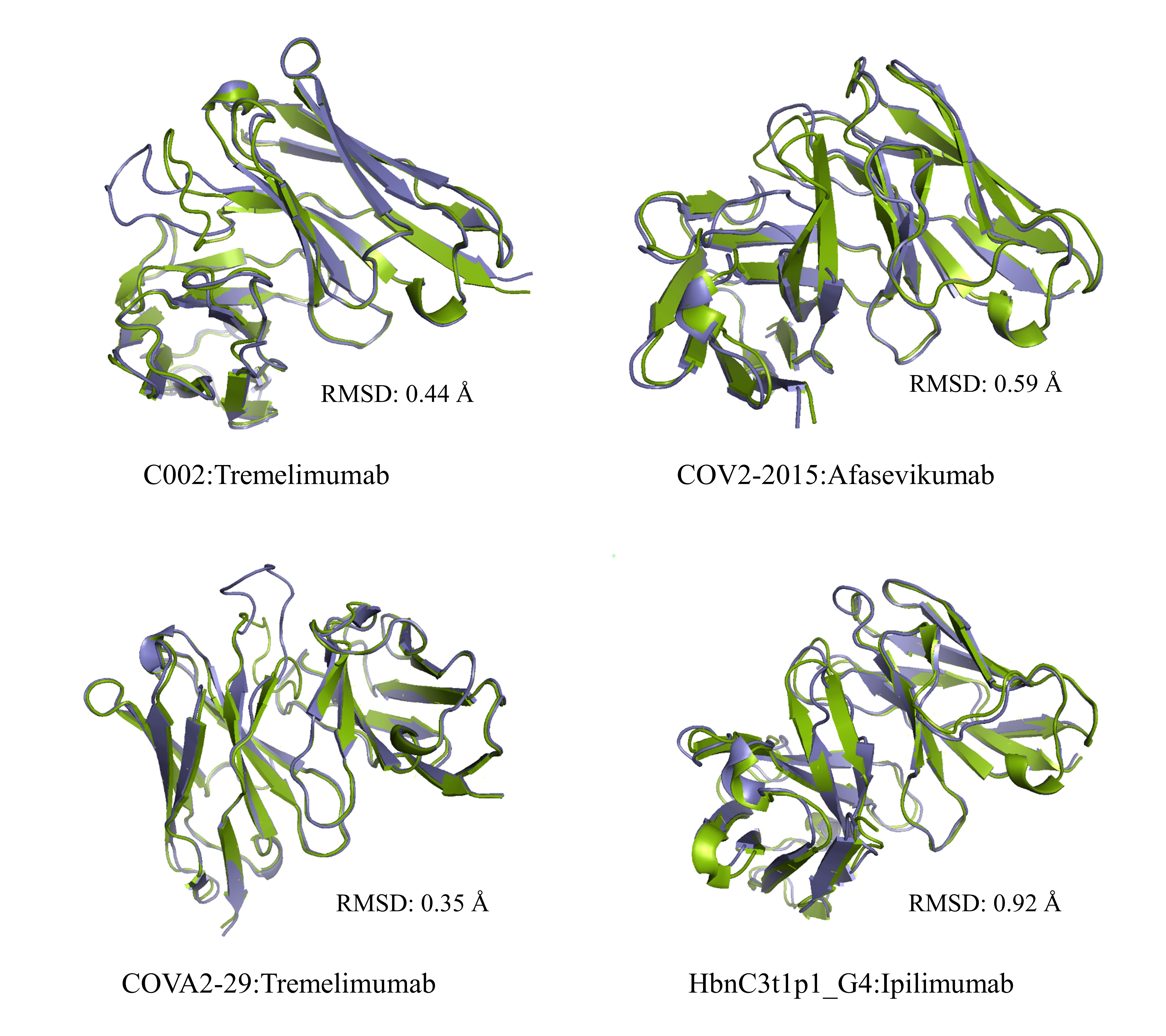


**Figure S4.** Relative positions of the regions considered as epitopes and ACE2 binding site (green) on the RBD region of spike protein (PDB id: 6M0J, blue). Mesh denotes the surface of the RBD region. Details on amino acid residue positions are presented in Table S4. The figures are generated using PyMOL 2.4 (https://pymol.org/2/).


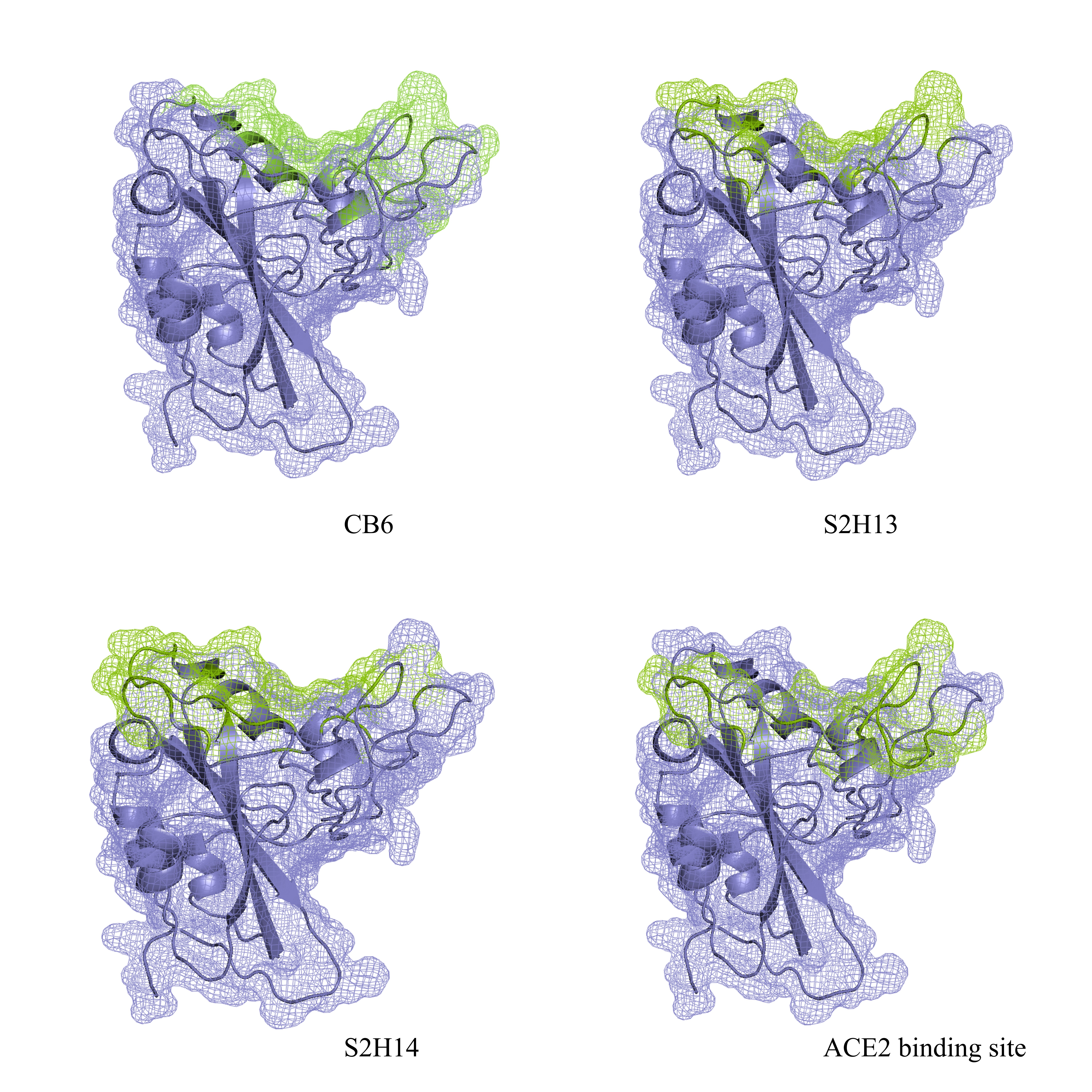


**References**

Piccoli, L., Park, Y. J., Tortorici, M. A., Czudnochowski, N., Walls, A. C., Beltramello, M., et al. (2020). Mapping neutralizing and immunodominant sites on the SARS-CoV-2 spike receptor-binding domain by structure-guided high-resolution serology. Cell 183, 1024-1042.

Li, Y., Lai, D. Y., Zhang, H. N., Jiang, H. W., Tian, X. L., Ma, M. L., et al. (2020). Linear epitopes of SARS-CoV-2 spike protein elicit neutralizing antibodies in COVID-19 patients. Cell. Mol. Immunol. 17, 1095–1097.
